# Developmental origins of non-communicable disease: Implications for research and public health

**DOI:** 10.1186/1476-069X-11-42

**Published:** 2012-06-27

**Authors:** Robert Barouki, Peter D Gluckman, Philippe Grandjean, Mark Hanson, Jerrold J Heindel

**Affiliations:** 1 INSERM UMR-S 747, Université Paris Descartes, Paris, 06, 75270, France; 2 University of Auckland, Private Bag 92019, Auckland, 01142, New Zealand; 3 Environmental Medicine, University of Southern Denmark, Odense, 5000, Denmark; 4 Harvard School of Public Health, Boston, MA, 02215, USA; 5 University of Southampton, Mailpoint 887, Southampton General Hospital, Southampton, SO16 6YD, UK; 6 National Institute of Environmental Health Sciences, P O Box 12233, Research Triangle Park, Durham, NC, 27709, USA

**Keywords:** Environmental exposure, Fetal development, Non-communicable disease, Nutritional requirements, Prenatal exposure delayed effects

## Abstract

This White Paper highlights the developmental period as a plastic phase, which allows the organism to adapt to changes in the environment to maintain or improve reproductive capability in part through sustained health. Plasticity is more prominent prenatally and during early postnatal life, *i.e.*, during the time of cell differentiation and specific tissue formation. These developmental periods are highly sensitive to environmental factors, such as nutrients, environmental chemicals, drugs, infections and other stressors. Nutrient and toxicant effects share many of the same characteristics and reflect two sides of the same coin. In both cases, alterations in physiological functions can be induced and may lead to the development of non-communicable conditions. Many of the major diseases – and dysfunctions – that have increased substantially in prevalence over the last 40 years seem to be related in part to developmental factors associated with either nutritional imbalance or exposures to environmental chemicals. The Developmental Origins of Health and Disease (DOHaD) concept provides significant insight into new strategies for research and disease prevention and is sufficiently robust and repeatable across species, including humans, to require a policy and public health response. This White Paper therefore concludes that, as early development (*in utero* and during the first years of postnatal life) is particularly sensitive to developmental disruption by nutritional factors or environmental chemical exposures, with potentially adverse consequences for health later in life, both research and disease prevention strategies should focus more on these vulnerable life stages.

## Background

For many years biologists considered the developmental period to be controlled by a strict, hard-wired genetic program, and thus it was uncertain how it could be influenced by the environment. It is now clear that development is plastic, and that it allows the organism to respond to the surrounding environment, especially during early development when cells are differentiating and tissues are developing. This capacity is based on molecular pathways that lead to control of gene expression and induction of specific phenotypes in the absence of DNA sequence modification [1]. These pathways, as currently understood, include DNA methylation, histone covalent modification, and noncoding RNA expression. Such epigenetic modifications can be passed from one cell generation to the next and, in some cases, when germ cells are targeted, can be transgenerationally transmitted [2]. Furthermore, these changes can be cell, tissue, and sex specific, and time dependent. In many cases they may not be apparent during a latent period which may last from months to years or decades. Thus, each individual has one genome, but will hold multiple epigenomes.

The ability to respond to environmental conditions can be evolutionarily advantageous by allowing fine-tuning of gene expression, likely through epigenetic mechanisms [3]. Thus, developmentally plastic processes allow the organism to adapt to changing environments in order to maintain or improve reproductive capability in part by sustaining health through the reproductive period. However, interference with these developmentally-adaptive processes may also have adverse consequences on some functions and disease risks later in life. Furthermore, these mechanisms are also sensitive to environmental stimuli other than the nutrients and physiological factors that are normative, in evolutionary terms, to the human environment. Indeed, drugs, industrial chemicals, tobacco smoke, and other environmental exposures can affect these same mechanisms leading to adverse consequences such as increased disease risk [4].

The sensitivity of the epigenetic system to environmental factors occurs primarily during the period of developmental plasticity because this is the time when epigenetic marks undergo critical modifications [5]. Once a tissue or system is fully developed, while still somewhat plastic, it is less sensitive to alterations by environmental stimuli. The most sensitive window for epigenetic effects is different for each tissue and may extend into early childhood and perhaps into puberty or beyond for some tissues such as the brain and the reproductive system [4,6]. There are also sex differences in the effects on gene expression and disease risk. Clearly, epigenetic alterations provide remarkable molecular candidate mechanisms even for subtle developmental toxicity to cause delayed effects. However, such mechanisms still need further experimental exploration.

Since nutritional imbalance (over- or under-nutrition) or environmental chemical (toxicant) exposures during development can each increase disease risk, their effects are likely to share some common pathways. Indeed, hormones, cytokines and nutritional components can both directly activate receptors that stimulate gene expression and also activate or inhibit the enzymes and pathways that are responsible for DNA methylation, chromatin remodelling and non-coding RNAs which ultimately control gene expression. Thus, epigenetic regulatory pathways are likely sites for effects of both nutrient and environmental toxicant effects during development and potentially also across the lifespan.

In this White Paper, we highlight key features of this paradigm and indicate research and policy changes needed to develop a stronger focus on prevention of non-communicable conditions. A draft was developed for discussion at the PPTOX III conference on Environmental Stressors in the Developmental Origins of Disease: Evidence and Mechanisms, held in Paris, France on 14–16 May, 2012. This revised version takes into account written comments submitted by conference participants. In the spirit of the Faroes Statement [7], this White Paper aims at providing an updated overview to outline the new research challenges and the possible implications for public health. However, it cannot provide an extensive list of references from this burgeoning field and refers only to selected review articles [1-6,8].

The conditions that are affected by nutritional or environmental chemical exposures during development include the pathophysiologies, diseases, and syndromes that constitute major public health problems across the globe: obesity, diabetes, hypertension, cardiovascular disease, asthma and allergy, immune and autoimmune diseases, neurodevelopmental and neurodegenerative diseases, precocious puberty, infertility, some cancer types, osteoporosis, depression, schizophrenia and sarcopenia.

## The risk of many non-communicable diseases is set during development

Advances in our understanding of molecular epigenetics have added considerable insight into the effects of environmental stimuli during development and the significance of the timing of exposures to these stimuli on later human health. It provides mechanisms for observations that were initially descriptive. All complex diseases have an environmental component, and a shrinking fraction is attributed solely to fixed genetic variation. This conclusion is based on the substantial increase in incidence of many non-communicable conditions during the last 20–40 years, a time interval much too short to be attributable to genetic change. The scientific focus has therefore turned to gene-environment interactions as a dominating contributor to disease susceptibility and pathogenesis. To prevent such disease or dysfunction, the interaction of genetic and environmental components must be explored across the lifespan, though with a new and strengthened focus on early development.

For the purposes of this White Paper, “environment” comprises nutrition, infections, the microbiome, drugs, man-made environmental chemicals, and other exogenous stresses. Some exposures are largely novel (in evolutionary terms) and anthropogenic, and so their effects, and the physiological responses to them, have not been subject to selection pressure. In contrast, the human body has evolved mechanisms of developmental plasticity and homeostasis to cope with changes in nutrition, stress, infections and the microbiome as commonly encountered modifiers and stressors. Whether the resultant change in phenotype from this latter group is adaptive (*i.e.*, might confer a fitness advantage) depends on the nature and strength of the stimuli during development and whether they convey useful information about the external environment later in life. If there is a match between the adaptive change and the subsequent lifetime environment then the change is likely to be beneficial to adult health. If there is a *mismatch* between the adaptive change and the subsequent lifetime environment then the response will likely be in fact maladaptive and lead to dysfunction and greater risk of disease. However, it is important to note that changes can be adaptive for certain endpoints, but maladaptive for others, as in the case of energy storage as fat during development which is beneficial and pathological obesity in adulthood. Other components of the life-course strategy interact, as in the trade-off between defence and repair processes up to the time of reproduction, with a decline during aging. It is important in this context to note the marked increase in lifespan in recent decades which exposes situations where natural selection has been weak or absent.

Disruption of normal signalling pathways during development occurs if the challenge is very strong or evolutionarily novel. It can result in gross effects, such as death, birth defects, and low birth weight. However, more commonly, the functional changes are subtle, at least initially. Thus, developmental exposures to environmental chemicals at low doses, especially endocrine disrupting chemicals (EDCs), can result in functional changes in gene expression, and whilst they do not lead to any phenotype change observable at birth they nonetheless may lead to increased risk of dysfunction and disease later in life. In contrast, exposure to environmental chemicals in adults typically results in acute effects that persist only as long as the environmental chemical is present. Still, other sensitive windows may occur, *e.g.*, during pregnancy, pre-puberty and aging, so caution is required when interpreting results from exposure paradigms beyond early development.

Although the term disruption is widely used in the context of EDCs and is retained here, its use in regard to environmentally-induced developmental changes in general implies that normal developmental processes may not always be disrupted. Thus, nutritional signals, EDCs and other agents can act on endogenous receptors (*e.g.*, steroid hormone receptors), and thereby affect normal control processes, although not necessarily inducing a pathological state.

## Nutritional imbalance during development can increase risk for disease later in life

The underlying causes of fetal under-nutrition worldwide include: poor or unbalanced maternal nutrition; suboptimal body composition; excessive physical workload before and during pregnancy; and poor function of the fetal supply line (*e.g.*, placental dysfunction). Maternal under-nutrition remains a major problem in low- and middle-income countries. Inadequate and poor quality diets are more common among women of low income and low educational attainment, in addition to those who are food insecure. While current evidence of fetal influences on later health has been mainly linked to maternal diet, emerging evidence points to the important role of maternal obesity, excessive weight gain in pregnancy, and gestational diabetes as factors that influence disease risks in the next generation.

Evidence for the effects of maternal malnutrition on offspring comes from a historical cohort of Dutch individuals whose mothers were exposed during the wartime famine of 1944–1945. Offspring of women exposed during early pregnancy were more likely to develop the metabolic syndrome in adulthood compared to offspring of women pregnant before or after the famine. The effects were dependent on the trimester of gestation in which famine was experienced. Epigenetic analyses in these individuals nearly 60 years later show differential methylation in several genes involved in growth and metabolic control, which are dependent on sex and time of exposure during gestation. Hypomethylation at the promoter of *IGF2*, a maternally imprinted gene implicated in growth and development, has also been observed in those exposed during the peri-conceptional period relative to unexposed siblings, although the effect is small. In other more recently established cohorts, individuals exposed *in utero* and infancy to the Nigerian civil war famine of 1968–70 were at increased risk of hypertension, impaired glucose tolerance and being overweight about 40 years later. Similarly, women exposed to the 1959–61 famine in China during gestation or early childhood are reported to have a greater risk of metabolic syndrome.

Recent studies have shown that even subtle imbalances in maternal nutrition are associated with the epigenetic profile at birth, which in turn is linked to markers of metabolic risk. Maternal carbohydrate consumption during the first trimester of pregnancy is inversely correlated with methylation levels at a single CpG in the *RXRA* gene in umbilical cord tissue and, in turn, is associated with child’s adiposity at age 6 or 9 years of age.

The early epidemiological data showed that the relation between birth weight and later disease risk is U-shaped; high birth weight also is associated with greater disease risk. This effect, although small in historical cohorts such as the Hertfordshire study, is very pronounced in populations such as the Pima who have a high prevalence of metabolic disease. There is now increasing epidemiological evidence that fetal overnutrition – as judged from indicators such as maternal obesity, excessive gestational weight gain, and gestational diabetes (GDM) – can produce a similar offspring phenotype to that of undernutrition. For example, maternal body mass index (BMI) has been positively correlated with total and abdominal adiposity, and with hepatic lipid content in infancy, across the entire range of maternal BMI. Exposure to a diabetic intrauterine milieu is a main risk factor for type 2 diabetes and is also associated with greater adiposity from infancy. Pregnancy weight gain that exceeds the medical guidelines is also associated with higher neonatal and early adult obesity. The epigenetic basis for these effects is now being explored.

The dramatic increases in rates of overweight, obesity, and GDM in pregnant women during the past decade may have serious long-term consequences for public health. In many western countries, close to half of all expectant mothers are now overweight, and the prevalence of GDM in some Asian countries has increased to 20%. In Canadian First Nation populations, GDM may explain up to 30% of the highly increased incidence of type 2 diabetes in the next generation. These trends are generating a vicious trans-generational cycle of ‘diabesity’.

## Exposures to environmental chemicals during development can increase disease risk later in life

While congenital malformations were long thought to be mediated by fixed genetic mutations, the phocomelia caused by thalidomide changed the perception that human development is autonomous. Fetal alcohol syndrome and the impact of congenital rubella infection further added to the evidence that stressors could result in lasting adverse effects. In all of these cases, the exposure occurred *via* the pregnant mother, who was herself barely affected, and only during a short period of time. Thus, exposures that had virtually no detrimental effect on the mature organism caused very serious adverse effects on the developing fetus.

Teratological studies of birth defects originally focused on miscarriage, fetal death or pregnancy loss, birth defects and low birth weight. Teratogens were often mutagenic and caused changes in the DNA sequence, while others caused general toxicity leading to cell death. Higher doses were usually associated with more severe effects. In laboratory studies, the animals were usually killed at birth to allow assessment of possible birth defects, so that no follow-up was available to determine any possible impact as to disease or dysfunction later in life. Later on this practice was deemed lacking, when evidence surfaced that certain cancers may originate prenatally, nervous system functions may be affected by nutrients and toxicants during early development, and reproductive development can be harmed by drugs and toxicants causing endocrine disruption.

**Figure 1:**
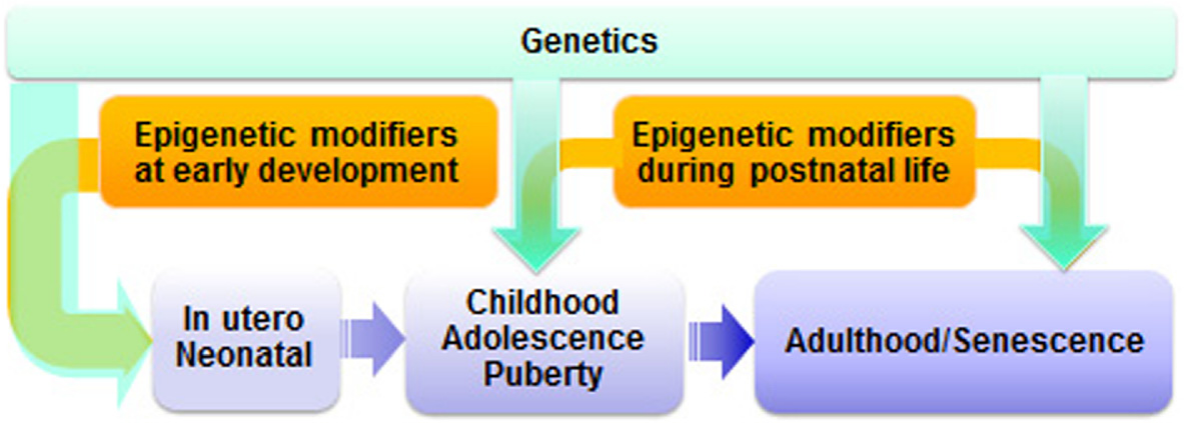
**Periods of vulnerability to environmental influences.** The most critical period is the perinatal period, during which epigenetic plasticity is high and can be influenced by a variety of environmental cues, including chemicals, nutrition, infection, *etc.*). Later in life, growth and the hormonally active puberty period is also a vulnerability period. In adults it is believed that elder persons are more vulnerable to a variety of insults

Further environmental chemical studies showed that exposures during development at low doses did not cause any teratogenic endpoints, although dysfunctions and diseases showed up later in life. In addition, the chemicals affected tissue and organ functions only when the exposure covered critical windows of development. This observation is consistent with emergent concepts in developmental physiology. As tissue development is controlled by epigenetic processes, which, in turn, are influenced by hormones and growth factors with which environmental chemicals such as EDCs can interfere. In some tissues and organs, such as the brain, lungs and immune system, developmental vulnerability continues through the neonatal period and perhaps into puberty, thereby extending the period of increased vulnerability to adverse effects from environmental chemicals. The original concept of a fetal basis of adult disease was therefore changed to that of developmental origins of adult disease, more recently modified to Developmental Origins of Health and Disease (DOHaD), as adverse effects may already emerge in childhood and adolescence and health-disease are part of a continuous spectrum of outcomes in response to risk factors (Figure 1).

While EDCs initially referred to substances interfering with reproductive hormones, the term now extends to compounds that may affect any endogenous hormones that carry signals from one cell to another, and there are now about 900 chemicals characterized as EDCs. Such compounds can alter the effects of the endogenous hormones by acting as receptor agonists or antagonists (or both, when acting as modulators), thereby resulting in abnormal hormonal signalling and leading to altered hormone action. EDCs can also act by affecting hormone concentrations indirectly through signalling pathways that control hormone production or elimination. Binding of EDCs to hormone receptors results in tissue-specific, hormone-like effects that may occur at low doses with dose–response relationships that may be non-monotonic (*e.g.*, U-shaped or other non-linear) where the effects at low doses can be more harmful than at higher doses. For this reason, the effects of low EDC doses cannot be predicted by extrapolation from high dose testing results. Such non-monotonic dose–response curves are well known for many normal physiologic mechanisms that may include activation of different pathways at different dose levels and down-regulation of receptors at high hormone or EDC concentrations.

**Figure 2:**
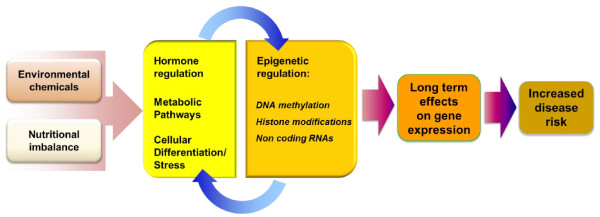
**Common mechanisms of nutritional disturbance and environmental chemicals.** Both nutritional unbalance and exposure to environmental chemicals can alter hormonal regulation, metabolic pathways, cellular plasticity and a variety of stress signals such oxidative and endoplasmic reticulum stress. These influence epigenetic factors such as DNA methylation, histone post translational regulation and noncoding RNA expression. However, these epigenetic effects in turn alter cellular and physiological pathways which may exacerbate their effects, ultimately leading to long term effects and children or adult diseases

Biomonitoring studies have shown that humans are exposed to hundreds of environmental chemicals, many of which are EDCs. Some examples of EDCs known to alter disease susceptibility as a result of developmental exposures in animal models include bisphenol A (from polycarbonate plastics), phthalates (a softener in plastics), some organophosphate and organochlorine pesticides, nicotine (tobacco smoking), air pollution, perfluorooctane compounds (stain and water repellents), and polybrominated diphenyl ethers (flame retardants) – all chemicals that are found in detectable concentrations in blood or urine samples from most people. As with nutrient effects during development, several EDC effects appear to affect specific genes due to alterations in epigenetic marks. Importantly, changes in gene expression resulting from EDC exposures during development may not be detectable at birth, and some will only show up later in life. Thus, changes in epigenetic marks may be useful biomarkers of exposures and potential disease risk later in life (Figure 2).

## Developmental nutrient and toxicant exposures: Different sides of the same coin

The concept of adult disease having a fetal basis started with a focus on severe malnutrition during pregnancy in humans and the susceptibility during adulthood to type 2 diabetes, high blood pressure, and cardiovascular disease, indicated by a range of early studies, and later expanded by David Barker and colleagues. Concurrently with those studies, the adverse health effects of diethylstilboestrol (DES), an estrogenic drug given to pregnant women to protect pregnancy loss, became apparent in the offspring of both sexes, thus indicating that maternal exposures had consequences that affect the health of their children. Howard Bern coined the term “the fragile fetus” to indicate fetal vulnerability to endocrine disrupting chemicals. This was followed by the experimental demonstration that developmental exposure to environmental chemicals in laboratory animals could lead to increased susceptibility to disease and dysfunction later in life. While these two fields of science started independently, it is now clear that both are reflections of the life-course implications of the developmental environment. Both nutritional imbalance and environmental chemical exposure, during sensitive windows of development, act when tissues are forming, to affect the phenotype, thereby impacting on organ functions and disease susceptibility later in life. So while the fetus may not only be ‘fragile’, it is plastic and is able to respond adaptively to a wide range of challenges and stimuli during development. Nonetheless this plasticity can set the scene for enhanced risk of disease later in life.

Both scientific areas are presently supported by extensive laboratory animal and human studies. Predisposition to many diseases has been shown to result from, or at least to correlate with, early changes in nutrition or chemical exposures. The two related disciplines are now starting to be integrated both in terms of basic and clinical science, and in terms of their public health implications, as nutrients and environmental chemicals can act on the same developmental systems over the same time frames and *via* similar mechanisms. In some cases, one factor may compensate for the effects of another, or the two may act synergistically to generate a stronger impact. The following features are common to both fields.

· Both nutritional imbalance and environmental chemicals act during specific windows of sensitivity and thus show time-, sex-, and tissue-specific effects. The windows of sensitivity are known only in general terms, with most research focused on the *in utero* and early childhood time periods. The effects on disease risk are likely to be due to multiple impacts by nutritional challenges and chemical exposures accumulated from embryonic development throughout the life-course. These may include early childhood, puberty, pregnancy, menopause and aging (Figure 1).

· While the adverse effects of nutritional imbalance during development can result in diseases and dysfunctions later in life, some effects are mediated by adaptive changes that protect the fetus and child, but are to the long-term detriment of specific tissue and organ function, thereby resulting in increased susceptibility to disease, depending on the differences between neonatal and adult environments. Similarly, exposure to environmental chemicals during development can cause abnormal gene regulation (*e.g.*, *via* epigenetic mechanisms) which may persist and may become apparent later in life as increased risk of dysfunctions or diseases (Figure 2). Thus, developmental disruption can result from multiple impacts by nutritional imbalance and chemical exposures, accumulated from embryonic development throughout the life-course.

· Neither nutritional imbalance nor chemical exposures need to affect birth weight to generate a longer-term effect on disease risk. Indeed, there is a continuum of risk even among those within the normal range of nutrition (and birth weight) and with very low doses of environmental chemicals. For chemical exposures, the effects are most often not accompanied by a clear change in birth weight. Thus, new biomarkers, for example, epigenetic parameters, at birth are needed to assess the potential for increased disease risk.

· The changes that occur during development due to nutritional imbalance or environmental chemicals are often functional in nature, and include alterations in gene expression, protein concentrations, cell metabolism and differentiation, and in cell numbers or location, thus affecting interactions between cell types and the establishment of cell lineages. These changes can lead to subtle morphological changes detected only during detailed histologic examination and/or changes in the functional characteristics of tissues, organs or systems. However, the effects of nutrient imbalance or chemical exposure need not necessarily be functional at birth: epigenetic changes in particular can produce permanent effects on the promoter regions of specific genes, which will not become apparent until the appropriate stimuli for expression, *e.g.* levels of transcription factors, are present. This emphasizes again the need for effective biomarkers, especially epigenetic marks, which can be measured at birth and which not only indicate responses to prenatal challenges, but also potential later responses to risk factors for disease.

· Functional changes result in changed susceptibility to non-communicable diseases that will likely show up later in life, with a latency that may vary from months to years or even decades. The disease or functional outcome will depend on the stressor, its concentration and timing. Again, the latency before the appearance of health impacts necessitates the development of biomarkers of exposure and the future risk of ill health that can be measured early in life.

· A combination of developmental stressors, whether nutritional or toxic, could cause effects jointly with similar or other exposures at different times to trigger or exacerbate adverse effects. While such combinations may result in additive effects on similar pathways, cells and tissues, the details vary. The adverse effects resulting from such stresses can become apparent as an increased incidence of a disease or dysfunction, an earlier onset or an increased severity of the trait. However, the possibility exists that some interactions between nutrients and toxicants may lead to *reduced* disease risk.

· The effect of either nutritional imbalance or environmental chemical exposures, depending on the dose and timing of exposures, can be transmitted *via* the germ line to subsequent generations, thus resulting in transgenerational inheritance of increased disease risks.

· The major mechanism, as currently understood, involves epigenetic processes, *i.e.*, altered DNA methylation, chromatin remodelling and small non-coding RNAs. The overall result is differential modulation of epigenetic systems that control gene expression that persists through mitosis.

· The effects of these stressors can also depend on genetic background, *e.g.* genetic polymorphisms. The effects may be sex-specific and may include changes in normal sexual dimorphism.

## Non-communicable diseases and dysfunction associated with nutritional and/or environmental chemical exposures

Developmental processes affect the risk of a wide range of important non-communicable diseases and can partly explain the increased prevalence of important diseases over recent decades. Diseases that have serious implications for public health include heart disease, obesity and type 2 diabetes, certain cancer types, and dysfunction of the reproductive, neurocognitive and immune systems, all of which may have a substantial economic and societal impact.

One important example is obesity that has globally increased in prevalence. Feeding behaviour, satiety, energy metabolism, and glucose/insulin sensitivity are examples of a complex interacting control system that involves the brain, adipose tissue, the gastrointestinal tract, muscle, liver, and the pancreas, all of which are connected by neurohumoral processes, and for which the integrated control is established in part during early development.

Unbalanced nutrition *in utero*, associated with either under- and over-nutrition, can result in increased rates of obesity later in life. A high maternal pre-pregnancy BMI has been linked to increased gestational weight gain, with increased birth weight and fat mass at birth. Maternal gestational diabetes is also associated with increased birth weight as well as childhood risks of overweight and obesity and, later, of diabetes. Interestingly, paternal nutrition has also been linked to adverse health outcomes in offspring in animal models, *e.g.*, paternal obesity can lead to disrupted insulin secretion and glucose tolerance in offspring. Furthermore, the developmental disruption can be subtle: maternal nutritional status can affect the offspring epigenetic states and body composition development independently of birth weight; first-born children are more likely to develop obesity and hypertension as adults in part because of greater maternal constraint in first pregnancies slightly reducing fetal growth and thus leading to greater potential mismatch.

Exposure to environmental chemicals has also been shown to result in increased risk of obesity later in life. These chemicals are referred to as obesogens. There are now about 20 chemicals and chemical classes that are known to lead to increased risk of weight gain later in life after developmental exposure and many of these same chemicals can also increase insulin resistance leading to type 2 diabetes, either concurrently with the obesity or independent of weight gain (examples include phthalates, bisphenol A, tributyltins, and several pesticides).

Other data indicate that developmental exposures to environmental chemicals can interact with unbalanced nutrition leading to aspects of metabolic syndrome later in life, indicating that both nutrient and chemical exposures can affect epigenetic processes controlling weight gain, metabolism and glucose tolerance. Indeed the “perfect storm” for obesity could be an interaction between unbalanced nutrition and environmental chemical exposures during development altering the set-point for weight gain and metabolism *via* effects on adipose tissue deposition, pancreas, muscle, gastrointestinal tract, liver and brain functions. This altered metabolism could be continually worsened by mismatched environmental exposures throughout life along with high fat and sugar diets as well as insufficient exercise.

Development of the human reproductive system begins toward the end of the first trimester; studies have shown its sensitivity to environmental chemicals, especially EDCs. A variety of dysfunctions and diseases, such as cryptorchidism, low semen quality, subfecundity, polycystic ovarian syndrome, testicular cancer, and uterine fibromyoma, have been linked to developmental exposures to EDCs. Unbalanced nutrition and growth during development can also lead to changes in reproductive function, *e.g.*, the timing of puberty and fecundity.

Brain development involves complex developmental stages that must happen at a particular time and sequence. Disruption of a developmental process can lead to permanent damage that may be reflected in cognitive deficits, emotional or behavioural change. These changes may have important consequences in terms of lifetime income, delinquency risks or later development of degenerative nervous system disease. The mechanisms of induction of such adverse neurobehavioral outcomes are unclear and may involve neuron or progenitor cell toxicity, endocrine disruption, and other mechanisms brought about either by nutritional stressors or toxicant exposures. Epigenetic mechanisms relating developmental stress, nutrition and/or environmental chemicals, to behaviour and neuroendocrine function have been implicated in both animal and human studies.

The immune system undergoes important maturation during early postnatal development. As originally reflected in the so-called ‘hygiene hypothesis’, the recognition of foreign antigens after parturition allows proper detection of and protection against invading micro-organisms and against cells with an abnormal phenotype. If this developmental exposure does not happen optimally, adverse consequences may include allergy, autoimmune disease, inflammation and cancer. Again, the importance of developmental exposures has been documented, but not the specific role of nutritional imbalance or individual environmental chemicals, or their molecular mechanisms.

The examples above illustrate that developmental exposures to nutrients and environmental chemicals may have profound effects on organ functions and disease risks in later life. The risk factors also include tobacco smoke, drugs, and psychosocial stress. The overall impact of these factors, along with nutrients and environmental chemicals on the incidence of dysfunctions and non-communicable diseases, is likely to be substantial.

## Data gaps and challenges

The emerging scientific understanding of the long-term effects of developmental exposures adds a new dimension to the importance of preventing the negative effects of environmental chemicals and nutritional imbalance. This new research paradigm is challenging as it requires nutritionists, toxicologists, endocrinologists, developmental biologists, clinicians, and epidemiologists to look for causative factors in the past, as far back as each subject’s intrauterine life or even into the life of their parents or grandparents. It also demands that life science researchers using laboratory animals and other models consider early developmental stages as particularly vulnerable to external factors at relatively subtle levels and with effects which may only become manifest after periods of latency. The new insights also require prospective studies to define early-life exposures to chemicals and nutrients and provide a new emphasis on multi-generation studies.

To define in detail the role of nutrition and environmental chemical exposure during development in the etiology of disease will require team efforts in collaborative science. No one discipline alone can explore all the relevant aspects of disease etiology and pathogenesis from developmental biology to clinical diagnosis. A greater focus on mechanisms of the induction of disease risk is needed with an emphasis on early development.

Since human development is malleable because of developmental plasticity, the simple presence of epigenetic change is insufficient to predict disease. Thus, it is critical to identify those epigenetic changes that are most predictive of a later phenotype so that they can be used as relevant markers for disease prevention. As all human development involves a plastic element, the mere presence of epigenetic variation may not predict a significant effect on health in later life. Thus, a crucial research challenge is to identify the epigenetic modifications that are predictive of long-term adverse effects.

It is also clear that developmental nutritional imbalance or environmental chemical exposures can lead to more than one disease over the lifespan. Thus, a focus on one disease or one stressor may significantly underestimate the overall risk of these stressors to non-communicable disease and dysfunctions. Because disease prevention is the ultimate goal, these insights into disease pathogenesis will have important implications for researchers, funding agencies, regulatory agencies, as well as health care providers.

## Public policy implications

In order to control human exposure to causative substances, primary prevention and environmental interventions are required with a primary focus on early life, while employing a life-course approach to reduce the non-communicable disease burden and its associated impacts at personal, economic and social levels.

We therefore call for a shift toward primary prevention of disease based on the developmental origins of health and disease paradigm. A rational approach is to focus prevention measures on the mother-child pair during pre-pregnancy, pregnancy and the first few years of postnatal life. Measures which improve nutrition, and reduce exposures to environmental chemicals, from all environmental compartments (air, water, soil) and in food and consumer products, are likely to improve child and maternal health significantly over the short term, as well as reduce disease incidence and the cost of health care overall, thereby increasing the quality of life globally.

This focus on prevention in key developmental stages also necessitates a significant re-orientation of the education of healthcare professionals towards the developmental origins of health and disease.

Changes may be required in the way that risks related to environmental chemicals are assessed. While it is important to continue to identify those environmental chemicals that disrupt development leading to birth defects, there must be an increased effort to develop criteria and assays that will capture the latent effects on long term development reflected by the DOHaD paradigm. Current evidence on the importance of developmental disruption suggests that developmental testing should be included in all protocols for safety testing. Several aspects of developmental toxicity can already be examined using standardized protocols, and others must be added to cover the full spectrum of organ functions and disease endpoints that may be affected. While epigenetic marks may be important biomarkers both of exposures and of disease/dysfunction susceptibility, these endpoints need to be assessed and their usefulness in predicting risk determined.

If specific evidence is either absent or inconclusive, the DOHaD perspective suggests that risk assessments will need to include considerations on potential developmental disruption and its consequences so that prevention will not be unreasonably delayed because of scientific uncertainty.

## Conclusions

We believe that the developmental paradigm has reached the stage where the data, while not complete, are sufficiently robust and replicable across species, including humans, to require a policy and public health response. The current pandemic of non-communicable diseases and the increased prevalence of important dysfunctions demand an open interrogation of why current interventions appear insufficient. We now know that disease risk can be induced very early in the life course and that it is modifiable by nutrients and environmental chemical exposures (along with drugs. infections, and other types of stresses). The developmental disruption effects associated with nutrients and environmental chemicals are likely two sides of the same coin. These data provide clinicians and policy makers with pertinent information that can be used to develop procedures and policies which will lead to a reduction of the incidence of non-communicable disease.

A new approach towards disease prevention is needed, with a new emphasis on early development. A rational methodology is to improve nutrition and reduce environmental chemical exposures pre-pregnancy, during pregnancy and during the first few years of life. This change is likely to have a very large impact on reducing disease incidence and the cost of health care, while at the same time increasing the quality of life at a global level.

## Abbreviations

BMI, Body mass index; DES, Diethylstilboestrol; DOHaD, Developmental origins of health and disease; EDC, Endocrine disrupting compound; GDM, Gestational diabetes mellitus.

## Competing interests

RB is member of the scientific council of ANSES (France), PDG is science advisor to the Prime Minister of New Zealand, PG is a toxicology adviser to the Danish Board of Health, and JJH is an employee of the (US) National Institute of Environmental Health Sciences (NIH). Any statements, opinions or conclusions contained herein are the authors’ own and do not necessarily represent the statements, opinions or conclusions of their employers. The authors declare that they have no competing financial interests.

## Authors’ contributions

Each author drafted part of the first version of the manuscript, and all authors contributed to the revision. All authors read and approved the final manuscript.
